# Factors Associated with Ovarian Hyperstimulation Syndrome (OHSS) Severity in Women With Polycystic Ovary Syndrome Undergoing IVF/ICSI

**DOI:** 10.3389/fendo.2020.615957

**Published:** 2021-01-19

**Authors:** Bo Sun, Yujia Ma, Lu Li, Linli Hu, Fang Wang, Yile Zhang, Shanjun Dai, Yingpu Sun

**Affiliations:** ^1^Center for Reproductive Medicine, The First Affiliated Hospital of Zhengzhou University, Zhengzhou, China; ^2^Henan Key Laboratory of Reproduction and Genetics, The First Affiliated Hospital of Zhengzhou University, Zhengzhou, China; ^3^Henan Provincial Obstetrical and Gynecological Diseases (Reproductive Medicine) Clinical Research Center, The First Affiliated Hospital of Zhengzhou University, Zhengzhou, China; ^4^Henan Engineering Laboratory of Preimplantation Genetic Diagnosis and Screening, The First Affiliated Hospital of Zhengzhou University, Zhengzhou, China

**Keywords:** polycystic ovarian syndrome, ovarian hyperstimulation syndrome, body mass index, antral follicle count, estradiol

## Abstract

**Introduction:**

Age, polycystic ovary syndrome (PCOS), low body mass index (BMI), high antral follicle count (AFC), increased anti-Muller hormone (AMH) levels, and elevated serum estradiol (E2) concentrations are risk factors for ovarian hyperstimulation syndrome (OHSS). However, data on the relationship between risk factors and OHSS severity in patients with PCOS are rare.

**Objective:**

This retrospective study examined the risk factors for OHSS and their effect on OHSS severity in patients with PCOS undergoing *in vitro* fertilization (IVF)/intracytoplasmic sperm injection (ICSI).

**Method:**

The records of 2,699 women were reviewed and included in this study. These women were diagnosed with PCOS during their first IVF/ICSI cycle between January 2010 and December 2017. We analyzed the association between each of the interrogated risk factors (including female age, BMI, AFC, basal serum E2, and the number of oocytes retrieved) and OHSS. The effects of each risk factor on OHSS severity were further explored. Logistic regression was performed as part of the above analysis.

**Results:**

Of the 2,699 women with PCOS who underwent assisted reproductive technology (ART), 75.2% had a normal response to controlled ovarian hyperstimulation (COH), while 24.8% developed OHSS. All OHSS patients were younger and had lower BMIs and basal serum follicle-stimulating hormone (FSH) and E2 levels but higher AFCs than those in the normal group. AFC demonstrated a strong correlation with OHSS, with a cutoff value of 24 in patients with PCOS. A total of 19.5% of the patients had mild OHSS, while 80.5% had moderate OHSS. Compared with those in the moderate OHSS group, those in the mild OHSS group were older and had higher basal serum FSH levels and lower serum E2 and T levels. However, BMI and AFC were not different between the mild and moderate OHSS groups. Basal serum E2 showed a strong correlation with OHSS severity, with a cutoff value of 37.94 pg/ml.

**Conclusions:**

AFC is a strong marker of OHSS, and basal serum E2 is the best predictor of OHSS severity in women with PCOS undergoing IVF treatment.

## Introduction

Ovarian hyperstimulation syndrome (OHSS) is a complication that occurs frequently during controlled ovarian stimulation (COH) (1, 2). Moderate-to-severe OHSS occurs in 1%–5% of assisted reproductive technology (ART) cycles (3). OHSS is characterized by bilateral cystic enlargement of highly luteinized ovaries. Secondary complications, such as vascular hyperpermeability, which causes mortality in rare cases (4), as well as hemorrhagic ovarian cysts (5), also characterize OHSS. Because symptoms of the mild form of OHSS might go unnoticed, its morbidity is most likely underestimated. Moderate OHSS clinical manifestations include abdominal distention, nausea, and vomiting, as well as poor appetite. A total of 1.9% of patients are hospitalized because of effects that include hepatorenal failure, acute respiratory distress syndrome, hemorrhage from ovarian rupture, and thromboembolism. Severe OHSS cases may even result in death.

Previous studies have shown that polycystic ovary syndrome (PCOS), a history of OHSS, young age, low body mass index (BMI), and a high antral follicle count (AFC) might lead to OHSS. Other risk factors include a history of allergies, high levels of anti-Muller hormone (AMH), high doses of gonadotropins, and high serum E2 levels (6, 7). Following increases in human chorionic gonadotropin (HCG) in IVF/ICSI cycles, high serum E2 levels produced by ovarian follicles may cause over production of vascular endothelial growth factor (VEGF) and inflammatory factors (8, 9). Excessive VEGF and inflammatory factors cause dilatation of the vascular endothelium, which could cause a massive shift of body fluids into the interstitial space. PCOS is a common endocrine disorder, with a prevalence of 5%–8% in women of reproductive age (10–12), and it is a critical risk factor for OHSS. Patients with PCOS are characterized by hyperandrogenism, anovulation, and polycystic ovaries. Ovulation induction is a mainstay treatment for PCOS. Women with PCOS usually have higher AFCs and AMH and serum E2 levels. Due to the high sensitivity of polycystic ovaries to COH, controlling COH in patients with PCOS is difficult, and thus, COH may result in OHSS. A systematic review found an odds ratio of 6.8 for the development of OHSS with polycystic ovarian morphology (PCO) (13). OHSS has adverse effects on oocyte quality, embryo quality, and success (14–16). However, it remains controversial.

The current literature indicates that AMH values >3.4 ng/ml, AFC >24, and estradiol values >3,500 pg/ml are particularly associated with an increased risk of OHSS in patients undergoing fertility treatment (3, 17). However, the above indicators do not offer any further refinement or indications of the severity of OHSS. Previous studies have evaluated the risk factors for OHSS in the whole population being treated for infertility, especially non-polycystic ovary syndrome (NPCOS) patients, but the risk factors for OHSS in PCOS patients are still unclear. This retrospective study examines the risk factors for OHSS and their effect on OHSS severity in patients with PCOS undergoing IVF/ICSI treatment, which may help clinicians advance the management of patients with PCOS prevent the occurance of OHSS.

## Materials and Methods

### Study Design and Patients

A total of 50,518 records of female patients were reviewed in this study. A total of 2,699 women who attended the Center for Reproductive Medicine, The First Affiliated Hospital of Zhengzhou University from January 2010 to December 2017 were included (**Supplement Figure 1**). The participants had undergone the first *in vitro* fertilization (IVF)/intracytoplasmic sperm injection-embryo transfer (ICSI-ET) cycles using long agonist protocols and were diagnosed with PCOS based on the 2003 Rotterdam criteria. The diagnosis was based on at least two of the following three criteria: 1) Oligo/anovulation; 2) Clinical and/or biochemical signs of hyperandrogenism; or 3) Polycystic ovaries as well as exclusion of other etiologies (congenital adrenal hyperplasia, androgen-secreting tumors, Cushing’s syndrome) (18). Hyperandrogenism was determined based on the total serum T level. Polycystic ovaries were defined as ≥12 follicles of 2–9 mm in a single ovary or an ovarian volume of >10 cm3 on ultrasonography. Patients who underwent short ovarian stimulation protocols, had basal serum T level of >0.7 ng/ml and had severe systemic diseases, such as liver, cardiovascular, or kidney diseases, were excluded. Other exclusion factors included benign or malignant gynecological tumors (ovarian tumor, endometrial tumor, and cervical cancer), metabolic disorders, chromosomal abnormalities, endometriosis, and congenital uterine malformations.

### Grouping Method

Patients with PCOS were divided into two groups based on the presence of OHSS (normal group vs. OHSS group). And the OHSS group was divided into mild OHSS group and moderate OHSS group according to the following criteria.(since we do not have many severe OHSS patients in our center, we don’t discuss here). The diagnosis of OHSS was based on the 2016 published clinical practice guidelines, which highlight moderate abdominal pain, nausea, and vomiting as OHSS symptoms (17). Mild hyperstimulation was defined as bilateral ovarian enlargement, while moderate hyperstimulation was identified based on documentation of patient discomfort, bloating, ascites or nausea. Ascites (or pleural fluid), oliguria (<300 ml/d or <30 ml/h), hematopoietic cell transplantation (HCT)>0.45, hyponatremia (sodium <135 mmol/L), low osmotic pressure (osmotic pressure <282 mmol/L), hyperkalemia (potassium >5 mmol/L), hypoalbuminemia (serum albumin <35 g/L), and ovarian diameter >12 cm have been used to indicate severe condition (19, 20). Ultrasound was also used to confirm and classify OHSS as follows: mild OHSS: ovarian diameter <8 cm; moderate OHSS: ovarian diameter of 8–12 cm. Once a patient was diagnosed with OHSS, all embryos were cryopreserved.

### Ovarian Stimulation Protocols

All the included patients used long-acting protocol and they were given a starting dose of 87.5–225 IU human recombination follicle stimulating hormone (rFSH). Ovulation was induced using gonadotropin (Gn), and the dose was adjusted in a timely manner depending on the number, size, and growth of follicles. The clinician determined the increase or decrease based on hormone levels and follicular development. HCG injection depended on the size of one primary follicle (≥20 mm), with the diameter of the other follicle being ≥18 mm or more than 2/3 of the follicles having a follicle diameter ≥14 mm. The patient’s clinical serum E2 levels and follicle condition determined the trigger drug and HCG exposure time. Eggs were retrieved 36–37 h after HCG administration. Conventional IVF or ICSI was performed based on the semen quality and previous fertilization history. In summary, all patients were treated with a gonadotropin releasing hormone (GnRH) agonist protocol to achieve a receptive endometrium for the implantation of embryos. Intramuscular injection of progesterone was used for endometrial transformation. Luteal gel was used for luteal support after transplantation. HCG was not used.

### Statistical Methods

R version 3.4.4 (14) and SAS 9.4 (SAS Institute, Cary, NC, USA) were used to analyze the data. The numerical data are presented as the mean with standard deviation (SD), while categorical variables are shown as % (n/N). A t test was used for continuous variables, while the chi-square test was used for categorical variables. We performed logistic regression to explore the effects of each risk factor on OHSS and OHSS severity. The curve fit was used to clarify the relationships between OHSS and antral follicular count and basal follicle stimulating hormone (FSH). The results are reported as adjusted odds ratios (aORs) with 95% confidence intervals (CIs). Two-tailed *P*<0.05 was considered statistically significant.

## Results

### Baseline Characteristics

Of the 50,518 ART patients who began treatment between 2010 and 2017, 2,699 who had PCOS were included in the present study. There were 2,030 (75.2%) patients who had a normal response to COH, while 669 (24.8%) developed OHSS in response to COH (**Table 1**). The women in OHSS group were younger (29.12 ± 3.82 VS 28.60 ± 3.59), had a lower BMI (24.08 ± 3.57 VS 23.72 ± 3.54) as well as a lower basal serum FSH (5.94 ± 1.35 VS 5.59 ± 1.34) and E2 (46.92 ± 33.96 VS 43.99 ± 30.55) levels. On the other hand, OHSS patients showed a higher basal serum PRL (17.15 ± 12.50 VS 17.83 ± 11.30) and AFC (21.54 ± 4.34 VS 23.05 ± 2.60). As hypothesized, age, BMI or AFC were significantly different between the two groups (P = 0.0036; P = 0.0393; P < 0.0001), indicating that they might be important predictors of OHSS. And the basal serum E2 concentration differed from that in previous studies, which indicates that the level of basal serum E_2_ is predictive of OHSS severity.

**Table 1:** Baseline characteristics and hormonal profiles according to ovarian hyperstimulation syndrome (OHSS) status in women with polycystic ovary syndrome (PCOS) undergoing *in vitro* fertilization (IVF)/intracytoplasmic sperm injection (ICSI).

Variables	Normal	OHSS	*P* value
Number of patients	2030	669	
Age*	29.12 ± 3.82	28.60 ± 3.59	0.0036
Weight	62.80 ± 9.78	62.12 ± 9.90	0.1307
BMI*	24.08 ± 3.57	23.72 ± 3.54	0.0393
Duration of infertility	4.33 ± 2.22	4.23 ± 2.12	0.4428
Antral follicular count*	21.54 ± 4.34	23.05 ± 2.60	<0.0001
Baseline hormone levels			
FSH (mIU/ml)*	5.94 ± 1.35	5.59 ± 1.34	<0.0001
E2 (pg/ml)*	46.92 ± 33.96	43.99 ± 30.55	0.0338
P4 (ng/ml)	0.76 ± 0.75	0.77 ± 0.78	0.7538
PRL (ng/ml)*	17.15 ± 12.50	17.83 ± 11.30	0.0011
LH (mIU/ml)	8.70 ± 5.34	9.14 ± 5.56	0.0479
T (ng/ml)	0.37 ± 0.14	0.37 ± 0.14	0.6662
Type of infertility			
Secondary infertility	646 (31.82)	210 (31.39)	0.83485
Primary infertility	1384 (68.18)	459 (68.61)	
Treatment			
ICSI	417 (20.54)	106 (15.84)	0.00768
IVF	1613 (79.46)	563 (84.16)	
Proposal type			
Follicular phase long-acting protocol^#^	969 (47.73)	462 (69.06)	<0.0001
Long-acting protocol^#^	1061 (52.27)	207 (30.94)	

Data are mean ± standard deviation or N (% of response group). FSH, follicle-stimulating hormone; E2, estradiol; P4, progesterone; LH, luteinizing hormone; PRL, pituitary prolactin; T, testosterone; BMI, body mass index.

^#^They are both long agonist protocols, the difference between them is that the time of agonist-using, the former one is in the follicular phase, while the latter one is in the luteal phase.

*P < 0.05.

A total of 128 (19.5%) of the cycles involved mild OHSS, while 527 (80.5%) involved moderate OHSS. The baseline characteristics of the mild and moderate OHSS patients are listed in **Table 2**. Briefly, the mild OHSS group was older (29.42 ± 3.53 vs 28.41 ± 3.59, P=0.0027) and had a higher basal serum FSH level (5.89 ± 1.42 vs 5.48 ± 1.27, P=0.0026) and lower serum E2 (38.05 ± 30.90 vs 45.40 ± 29.61, P<0.0001) and serum T (0.34 ± 0.14 vs 0.38 ± 0.14, P=0.01) levels. Compared with mild OHSS patients, moderate OHSS patients were mostly treated with the follicular phase long-acting protocol (388/527, 73.62%). However, there was no difference in OHSS-related risk factors, such as BMI. In addition, there was no significant difference in the type of infertility or fertilization treatment between the mild OHSS and moderate OHSS groups.

**Table 2:** Baseline characteristics and hormonal profiles according to ovarian hyperstimulation syndrome (OHSS) severity in women with polycystic ovary syndrome (PCOS) undergoing *in vitro* fertilization (IVF)/intracytoplasmic sperm injection (ICSI).

Variables	Mild OHSS	Moderate OHSS	P value
Number of patients	128	527	
Age*	29.42 ± 3.53	28.41 ± 3.59	0.0027
Weight	61.89 ± 10.17	62.05 ± 9.83	0.7585
BMI	23.90 ± 3.76	23.65 ± 3.46	0.6651
Duration of infertility	4.54 ± 2.41	4.16 ± 2.04	0.2414
Antral follicular count	22.80 ± 2.84	23.13 ± 2.42	0.056
Baseline hormone levels			
FSH (mIU/ml)*	5.89 ± 1.42	5.48 ± 1.27	0.0026
E2 (pg/ml)*	38.05 ± 30.90	45.40 ± 29.61	<0.0001
P4 (ng/ml)	0.74 ± 0.73	0.77 ± 0.70	0.3422
PRL (ng/ml)	20.92 ± 29.50	19.95 ± 23.97	0.8363
LH (mIU/ml)	9.25 ± 6.07	9.03 ± 5.40	0.7007
T*	0.34 ± 0.14	0.38 ± 0.14	0.01
Type of infertility			
Secondary infertility	42 (32.81)	165 (31.31)	0.74283
Primary infertility	86 (67.19)	362 (68.69)	
Treatment			
ICSI	21 (16.41)	84 (15.94)	0.89723
IVF	107 (83.59)	443 (84.06)	
Proposal type			
Follicular phase long-acting protocol*^#^	62 (48.44)	388 (73.62)	<0.0001
Long-acting protocol^#^	66 (51.56)	139 (26.38)	

*P < 0.05.

Data are mean ± standard deviation or N (% of response group). FSH, follicle-stimulating hormone; E2, estradiol; P4, progesterone; LH, luteinizing hormone; PRL, pituitary prolactin; T, testosterone; BMI, body mass index.

^#^They are both long agonist protocols, the difference between them is that the time of agonist-using, the former one is in the follicular phase, while the latter one is in the luteal phase.

### Ovarian Induction Characteristics

Compared with the normal group, the OHSS group was associated with fewer starting dosages of Gn, a longer length of stimulation, more retrieved oocytes, a lower MII oocyte rate, and fewer good-quality embryos, as shown in **Table 3**. We further analyzed the above indicators by comparing them between the mild and moderate OHSS groups (**Table 4**). The mild OHSS patients were associated with more starting dosages of Gn (117.77 ± 25.90 vs 110.39 ± 21.12, P=0.0003), a shorter length of stimulation (13.66 ± 3.14 vs 14.22 ± 2.94, P=0.0258), fewer retrieved oocytes (19.40 ± 6.29 vs 23.65 ± 7.94, P<0.0001), a lower MII oocyte rate (46.61 ± 43.10 vs 64.60 ± 36.94, P=0.0002), and a higher rate of good-quality embryos (53.91 ± 22.77 vs 47.90 ± 21.74, P=0.0057).

**Table 3:** Ovarian stimulation characteristics according to ovarian hyperstimulation syndrome (OHSS) severity in women with polycystic ovary syndrome (PCOS) undergoing *in vitro* fertilization (IVF)/intracytoplasmic sperm injection (ICSI).

Variables	Normal	OHSS	*P* value
Number of patients	2030	669	
Starting dose of Gn (IU)*	125.11 ± 32.45	111.86 ± 22.27	<0.0001
Total amount of Gn (IU)	1941.78 ± 877.78	1969.69 ± 848.95	0.3127
Length of stimulation (d)*	13.25 ± 2.76	14.12 ± 2.98	<0.0001
Total oocytes retrieved*	15.31 ± 6.84	22.96 ± 7.93	<0.0001
Rate of MII oocytes*	69.18 ± 36.42	61.32 ± 38.76	<0.0001
Rate of 2PN embryos	75.24 ± 18.71	76.06 ± 17.04	0.6043
Rate of cleavage embryos*	98.79 ± 4.91	98.63 ± 3.97	<0.0001
Rate of good-quality embryos*	58.54 ± 27.86	49.06 ± 22.13	<0.0001
No. of ET cycles	1609 (79.26)	0	
Biochemical pregnancy rate	1139 (70.79)	–	
Clinical pregnancy rate	1067 (66.31)	–	
Live birth rate	918 (57.05)	–	

Numbers are mean ± standard deviation or N (% of response group). Gn, gonadotropin; MII, mature oocytes; 2PN, fertilized oocytes with two pronuclei; ET, embryo transfer.

*P < 0.05.

**Table 4:** Ovarian stimulation characteristics according to ovarian hyperstimulation syndrome (OHSS) severity in women with polycystic ovary syndrome (PCOS) undergoing *in vitro* fertilization (IVF)/intracytoplasmic sperm injection (ICSI).

Variables	Mild OHSS	Moderate OHSS	P value
Number of patients	128	527	
Starting dose of Gn (IU)*	117.77 ± 25.90	110.39 ± 21.12	0.0003
Total amount of Gn (IU)	1958.20 ± 889.87	1969.00 ± 841.82	0.6682
Length of stimulation (d)*	13.66 ± 3.14	14.22 ± 2.94	0.0258
Total oocytes retrieved*	19.40 ± 6.29	23.65 ± 7.94	<0.0001
Rate of MII oocytes*	46.61 ± 43.10	64.60 ± 36.94	0.0002
Rate of 2PN embryos	78.35 ± 17.35	75.73 ± 17.01	0.1698
Rate of cleavage embryos	98.52 ± 3.42	98.63 ± 4.14	0.3776
Rate of good-quality embryos*	53.91 ± 22.77	47.90 ± 21.74	0.0057

*P < 0.05.

Numbers are mean ± standard deviation or N (% of response group). Gn, gonadotropin; ET, embryo transfer.

### Hormone Level and Ovary Size During Controlled Ovarian Stimulation

The mild OHSS group had a smaller ovarian volume before and after HCG trigger (P<0.0001, left ovary size on HCG was 5.38 ± 0.85 vs 5.86 ± 0.95, P<0.0001, right ovary size on HCG was 5.89 ± 0.95 vs 6.38 ± 0.91; P<0.0001, left ovary size on HCG+3 7.00 ± 0.68 vs 8.55 ± 1.13, P<0.0001, right ovary size on HCG+3 7.04 ± 0.62 vs 8.80 ± 1.08). The moderate OHSS group had a much higher serum E2 level (P=0.0008, 2026.29 ± 730.84 vs 1790.31 ± 693.25) and thicker endometrium on HCG+3 (P=0.0386, 11.88 ± 2.21 vs 11.43 ± 2.15), as shown in **Table 5**. Meanwhile, there was a significant difference in the number of women with uterine effusion and cystocele on HCG+3 (P=0.00129, 23 vs 171) between the mild OHSS and moderate OHSS groups.

**Table 5:** Hormone level and ovary size during controlled ovarian stimulation.

Variables	Mild OHSS	Moderate OHSS	P value
Number of patients	128	527	
Endometrial thickness on HCG (mm)	11.54 ± 2.05	11.83 ± 2.12	0.1466
Serum E2 level on HCG (pg/ml)	6305.93 ± 2527.81	6195.29 ± 2688.66	0.4337
Left ovary size on HCG* (cm)	5.38 ± 0.85	5.86 ± 0.95	<0.0001
Right ovary size on HCG* (cm)	5.89 ± 0.95	6.38 ± 0.91	<0.0001
Endometrial thickness on HCG+3* (mm)	11.43 ± 2.15	11.88 ± 2.21	0.0386
Serum E2 level on HCG+3*(pg/ml)	1790.31 ± 693.25	2026.29 ± 730.84	0.0008
Left ovary size on HCG+3* (cm)	7.00 ± 0.68	8.55 ± 1.13	<0.0001
Right ovary size on HCG+3* (cm)	7.04 ± 0.62	8.80 ± 1.08	<0.0001
^Uterine effusion and rectocele on HCG+3* (mm)			0.09025
No	117 (91.41)	452 (85.77)	
Yes	11 (8.59)	75 (14.23)	
^Uterine effusion and cystocele on HCG+3* (mm)			0.00129
No	105 (82.03)	356 (67.55)	
Yes	23 (17.97)	171 (32.45)	

^the uterine effusion and recto-/cystocele are detected by transvaginal ultrasound, and the clinician is the same one on HCG = on the day of human chorionic gonadotropin administration.

*P < 0.05.

### Factors Associated With OHSS and OHSS Severity in Women With PCOS

Logistic regression analysis, incorporating conventional markers such as age, BMI, baseline hormone levels and AFC in the model, showed that only baseline hormone levels (FSH, E2, PRL, and LH) and AFC were significantly predictive of OHSS (**Table 6**). Among the patients with values less than the median values of the reference groups, the median values for FSH, E2, PRL, and LH were 5.74, 38.65, 14.95, and 7.02, respectively. Among all the variables analyzed, AFC was the best predictor of OHSS (OR [95% CI]: 1.87(1.51–2.32)), and the median AFC was 24 (**Supplement Figure 2**).

**Table 6:** Logistic regression analysis for OHSS.

	Beta	Standard Error	Wald Chi-Square Value	*P* value	OR (95% CI)
Age					
≤25 y					1.00 (Reference)
25~30 y	0.1877	0.0797	5.5501	0.0185	1.14(0.85–1.53)
30~35 y	0.00667	0.0928	0.0052	0.9427	0.95(0.69–1.31)
>35 y	-0.2508	0.1537	2.664	0.1026	0.74(0.46–1.17)
BMI					
18.5~23.9					1.00 (Reference)
23.9~27.9	-0.0437	0.0734	0.354	0.5519	0.85(0.69–1.04)
>27.9	-0.0782	0.0926	0.713	0.3985	0.82(0.62–1.09)
Antral follicular count*	0.6275	0.1098	32.64	<.0001	1.87(1.51–2.32)
Baseline hormone levels					
FSH (mIU/ml)*	-0.3937	0.0994	15.7017	<.0001	0.67(0.56–0.82)
E2 (pg/ml)*	-0.1447	0.0979	2.1865	0.1392	0.87(0.71–1.05)
PRL (ng/ml)*	0.2823	0.0972	8.4305	0.0037	1.33(1.10–1.60)
LH (mIU/ml)*	0.313	0.1024	9.3384	0.0022	1.37(1.12–1.67)
ICSI	-0.1103	0.0634	3.0246	0.082	0.80(0.63–1.03)
Long-acting protocol	-0.8048	0.1003	64.4436	<.0001	0.45(0.37–0.54)

*Select patients with less than the median value served as the reference group. The median value for the antral follicular count, FSH, E2, PRL, and LH was 24, 5.74, 38.65, 14.95, and 7.02, respectively.

FSH, follicle-stimulating hormone; E2, estradiol; P4, progesterone; LH, luteinizing hormone; PRL, pituitary prolactin; BMI, body mass index; ICSI, intracytoplasmic sperm injection.

**Table 7** shows a logistic regression analysis for OHSS severity. The basal serum FSH, E2, and T levels and AFC were significant predictors. Although these baseline hormone levels were equally predictive of OHSS severity, their ability to discriminate subjects who had a potential risk of OHSS severity was only of a modest degree (FSH (OR [95% CI]: 0.71(0.47–1.08)), E2 (OR [95% CI]: 1.61(1.04–2.47)), T (OR [95% CI]: 1.15(0.74–1.79)). Among the patients with values less than the median values of the reference groups, the median values for FSH, E2 and T were 5.49, 37.94, and 0.37, respectively. In addition, AFC was another predictive factor of OHSS severity (OR [95% CI]: 1.30 (0.80–2.10)), and the median AFC was also 24. Among all the variables analyzed, the basal serum E2 level was the best predictor of OHSS severity.

**Table 7:** Logistic regression analysis for ovarian hyperstimulation syndrome (OHSS) severity.

	Beta	Standard Error	Wald Chi-Square Value	P value	OR (95% CI)
Age					
≤25 y					1.00 (Reference)
25~30 y	0.1989	0.1738	1.3102	0.2524	0.79(0.39–1.60)
30~35 y	0.056	0.1995	0.0788	0.7789	0.69(0.33–1.46)
>35 y	-0.6846	0.3089	4.9123	0.0267	0.33(0.12–0.89)
Antral follicular count*	0.2607	0.2465	1.1179	0.2904	1.30(0.80–2.10)
Baseline hormone levels					
FSH (mIU/ml)*	-0.3403	0.2129	2.5557	0.1099	0.71(0.47–1.08)
E2 (pg/ml)*	0.4736	0.2206	4.6098	0.0318	1.61(1.04–2.47)
T*	0.1378	0.2264	0.3707	0.5426	1.15(0.74–1.79)
Long-acting protocol	-1.047	0.2122	24.3475	<.0001	0.35(0.23–0.53)

*Select patients with less than the median values served as the reference groups. The median value for the antral follicular count and FSH, E2, and T levels was 24, 5.49, 37.94, and 0.37, respectively.

FSH, follicle-stimulating hormone; E2, estradiol; T, testosterone.

## Discussion

Our study retrospectively analyzed 2,699 infertile women with PCOS who underwent their first ovarian stimulation cycles. During ovarian stimulation, PCOS patients with OHSS were younger and had lower BMIs, higher AFCs, and lower levels of basal serum FSH and E2. Moreover, PCOS patients with OHSS had more retrieved oocytes, a lower rate of MII oocytes and a lower rate of good-quality embryos. Only the AFC and basal serum levels of FSH and E2 were significantly correlated with OHSS. These results suggested that the AFC is the most important risk factor for OHSS in women with PCOS.

To further investigate the risk factors affecting OHSS severity, mild and moderate OHSS patients were compared. Compared with the moderate OHSS group, the mild OHSS group was older and had higher basal serum FSH and lower serum E2 and T levels. However, BMI and AFC were not different between the groups. Otherwise, the mild OHSS group had more starting doses of Gn, a shorter length of stimulation, fewer retrieved oocytes, a lower MII oocyte rate, and a greater number of good-quality embryos. As risk factors for OHSS, FSH, E2, and AFC were also significantly correlated with OHSS severity. The basal serum E2 level demonstrated the best correlation among them.

### Female Age and OHSS

Female age is a major factor influencing fertility. Increases in age reduce the reproductive capacity of women due to a reduced ovarian reserve, poor oocyte quality, and an increased prevalence of embryonic aneuploidy (21–23). Moreover, this study shows that age may also be used to assess the risk of OHSS and OHSS severity. Young women with high AFCs, high serum E2 levels and a high number of retrieved oocytes are more susceptible to OHSS. Two prospective studies and five retrospective studies that evaluated the effect of age on OHSS development showed that younger age was indeed a risk factor (24–28). Patient age has predictive significance for the occurrence of moderate-to-severe OHSS. The younger the patients are, the greater the possibility of developing severe OHSS. In our study of patients with PCOS, the OHSS group was younger, but age was not a risk factor for OHSS. The incidence rate of OHSS for patients aged between 25 and 30 years old, compared with those aged ≤ 25 years old, was increased (OR [95% CI]: 1.14(0.85–1.53)), but the severity of OHSS for the same age stratification was decreased (OR [95% CI]: 0.79(0.39–1.60)). This finding was probably because the patients in our study were generally young, which was inconsistent with previous studies.

### BMI and OHSS

BMI is another patient characteristic that was considered when assessing the risk of developing OHSS and OHSS severity. The initial Gn dosage for COH was determined based on patient characteristics, including BMI. A prospective observational cohort study showed that women with a BMI of ≥25 kg/m^2^ had significantly fewer mature oocytes, required a higher total dose of rFSH and had a higher risk of developing severe OHSS (29). However, a cohort study of 262 IVF cycles showed that BMI had no predictive value for OHSS (30). In contrast, our results showed that whereas there was no significant difference in BMI between OHSS patients with PCOS and patients with PCOS but no OHSS, OHSS patients had a lower BMI (23.72 ± 3.54 vs 24.08 ± 3.57). However, patients with mild OHSS and patients with moderate OHSS showed little difference in terms of BMI. Therefore, the influence of BMI on the development of OHSS and OHSS severity require further study.

### AFC and OHSS

Markers for ovarian reserve, especially serum AMH and AFC, may also be used to assess the risk of OHSS. The basal serum AMH level correlates well with the antral follicle count ultrasonographic findings (31). Regression analysis resulting from a retrospective study revealed that the AFC (OR, 95% CI; 4.3, 2.7–6.9) was the most important predictor of OHSS among non-polycystic ovary (NPCO) patients undergoing IVF/ICSI (24). In addition, Pelinalso *et al*. showed that AFC (AUC = 0.74) had moderate accuracy for predicting OHSS (32). Our study highlights that the mean FSH level was significantly lower (P < 0.0001) in women with OHSS than in those without OHSS. In a prospective analysis of 1,012 first ART cycles, the risk of OHSS increased from 2.2% in women with AFC < 24 to 8.6% in those with AFC≥24. Similarly, data from our study showed that the AFC cutoff value for OHSS in patients with PCOS was 24. In addition, AFC ≥24 correlated with an increased risk of moderate-to-severe OHSS. Compared with patients with mild OHSS, patients with moderate OHSS had a higher cutoff value (22.80 ± 2.84 vs 23.13 ± 2.42). Based on the data, our study shows that AFC predicts OHSS. Thus, knowledge of AFC levels is crucial in planning and managing the risks of over response and OHSS.

### Basal Serum E_2_, the Number of Oocytes Retrieved and OHSS

The development of OHSS is almost always accompanied by elevated estradiol (E2) levels, and estrogen has been implicated as a potential etiologic factor, one of the possible reason is that when the estradiol elevated, capillary permeability and the chemical mediators or precursors that augment fluid extravasation increased and thus moderate OHSS developed (33). The results from our study indicate that the level of basal serum E_2_ is predictive of OHSS, and the differences between the mild OHSS group and the moderate OHSS group have statistical significance (38.05 ± 30.90 vs 45.40 ± 29.61 P<0.001). Therefore, basal serum E2 showed a stronger correlation with OHSS severity and needs further study. A previous study showed that the number of retrieved oocytes is a risk factor for the occurrence of OHSS, and those with very high blood E2 levels and too many oocytes retrieved have an increased incidence of moderate-to-severe OHSS (34). In our study, the number of retrieved oocytes in the OHSS group was higher than that in the normal group (22.96 ± 7.93 vs 15.31 ± 6.84), but the rate of good-quality embryos was lower (49.06 ± 22.13 vs 58.54 ± 27.86). In addition, compared with the mild OHSS group, the moderate OHSS group generally had more oocytes retrieved. These results may help clinicians pay more attention to the basal serum E2 and the number of retrieved oocytes of patients with PCOS, which may prevent the occurance of OHSS.

### Strengths and Limitations

Our study revealed that AFCs have the most important impact on the occurrence of OHSS, especially in patients with PCOS. However, our study was conducted retrospectively and only involved a single medical center. In addition, serum AMH was measured in only a few patients, and thus these measurements were not included in this study. Finally, all patients undergoing ovarian induction were treated with a gonadotropin releasing hormone (GnRH) agonist protocol, weakening the generalizability of the findings.

In summary, AFC is an independent risk factor for OHSS in women with PCOS undergoing their first ovarian stimulation cycle. Besides, basal serum E2 is a predictive factor for OHSS severity. Meanwhile, patients with PCOS undergoing IVF/ICSI who are at risk of OHSS can be identified by careful ultrasonography of the ovaries before treatment. AFC >24 specifically is associated with an increased risk of OHSS, and the cutoff value is the same with NPCO patients, which indicates that patients with PCOS do not need the use of GnRH agonist cycles with low-dose FSH. This strategy will guide cycles of fresh embryo transfer instead of using a freeze-all strategy, improve the rates of live birth and lower the risks of OHSS.

### Database

The data were obtained from the ART electronic medical record system (CCRM) at the Reproductive Medicine Center of the First Affiliated Hospital of Zhengzhou University (a tertiary-care teaching hospital, Zhengzhou, China). All medical histories, ART treatment processes and the outcomes of every cycle are recorded in the CCRM. The records are validated by the Technical Standard for Human Assisted Reproduction issued by the Chinese Ministry of Health. The surveillance staff consults the patient information and performs quality control periodically.

## Data Availability Statement

The original contributions presented in the study are included in the article/**Supplementary Material**. Further inquiries can be directed to the corresponding author.

## Ethics Statement

The studies involving human participants were reviewed and approved by the Research Ethics Committee of the First Affiliated Hospital of Zhengzhou University.

## Author Contributions

BS and YM: designed study, analyzed data, and drafted the manuscript. LL: revised the manuscript. LH, FW, and SD: collected data. LH and YZ: reviewed the manuscript. YS: study conceptualization and review. All authors contributed to the article and approved the submitted version.

## Funding

This work was funded by the International (Regional) Cooperation and Exchange (ICE) Projects of the National Natural Science Foundation of China (NSFC) (FDN-81820108016), the Chinese Medical Association Clinical Medical Research Special Fund Project (FDN-17020190688), the Medical Science and Technology Research Project Joint Co-construction Project of Henan Province (FDN-2018020116), and the Henan Provincial Higher Education Key Re- search Project Plan (FDN-19A320056).

## Conflict of Interest

The authors declare that the research was conducted in the absence of any commercial or financial relationships that could be construed as a potential conflict of interest.

## Supplementary Material

Supplementary Figure 1 Flow chart of the patient selection process.

Supplementary Figure 2 Relationships between OHSS and the risk factors **(A)** antral follicular count and **(B)** basal FSH. **(A)** Adjusted for age, BMI, and basal FSH. **(B)** Adjusted for AFC, age, and BMI.

## References

[1] Luke B Brown MB Morbeck DE Hudson SB Coddington CC 3rd Stern JE Factors associated with ovarian hyperstimulation syndrome (OHSS) and its effect on assisted reproductive technology (ART) treatment and outcome. Fertil Steril (2010) 94:1399–404. 10.1016/j.fertnstert.2009.05.092 19591989

[2] Steward RG Lan L Shah AA Yeh JS Price TM Goldfarb JM Oocyte number as a predictor for ovarian hyperstimulation syndrome and live birth: an analysis of 256,381 in vitro fertilization cycles. Fertil Steril (2014) 101:967–73. 10.1016/j.fertnstert.2013.12.026 24462057

[3] Jayaprakasan K Chan Y Islam R Haoula Z Hopkisson J Coomarasamy A Prediction of in vitro fertilization outcome at different antral follicle count thresholds in a prospective cohort of 1,012 women. Fertil Steril (2012) 98:657–63. 10.1016/j.fertnstert.2012.05.042 22749225

[4] Nelson SM Prevention and management of ovarian hyperstimulation syndrome. Thromb Res (2017) 151(Suppl 1):S61–4. 10.1016/S0049-3848(17)30070-1 28262238

[5] Kurioka H Takahashi K Kita N Noda Y Hemorrhagic ovarian cyst without peritoneal bleeding in a patient with ovarian hyperstimulation syndrome: case report. Chin Med J (Engl) (2005) 118:1577–81. 16232339

[6] Corbett S Shmorgun D Claman P The prevention of ovarian hyperstimulation syndrome. J Obstet Gynaecol Can (2014) 36(11):1024–33. 10.1016/S1701-2163(15)30417-5 25574681

[7] Lee TS Liu CH Huang CC Wu YL Shih YT Ho HN Serum AMH and estradiol levels as predictors of OHSS in ART cycles. Hum Reprod (2008) 23(1):160–7. 10.1093/humrep/dem254 18000172

[8] Soares SR Gómez R Simón C García-Velasco JA Pellicer A Targeting the vascular endothelial growth factor system to prevent ovarian hyperstimulation syndrome. Hum Reprod Update (2008) 14(4):321–33. 10.1093/humupd/dmn008 18385260

[9] Liu M Xie S Zhou J Use of animal models for the imaging and quantification of angiogenesis. Exp Anim (2018) 67(1):1–6. 10.1538/expanim.17-0054 28757511 PMC5814308

[10] Norman RJ Dewailly D Legro RS Hickey TE Polycystic ovary syndrome. Lancet (2007) 370:685–97. 10.1016/s0140-6736(07)61345-2 17720020

[11] Brassard M AinMelk Y Baillargeon JP Basic infertility including polycystic ovary syndrome. Med Clin North Am (2008) 92:1163–92. 10.1016/j.mcna.2008.04.008 18721657

[12] Groth SW Adiponectin and polycystic ovary syndrome. Biol Res Nurs (2010) 12(1):62–72. 10.1177/1099800410371824 20498127 PMC3646519

[13] Mathur RS Tan BK British fertility society policy and practice committee: prevention of ovarian hyperstimulation syndrome. Hum Fertil (2014) 17(4):257–68. 10.3109/14647273.2014.961745 25380089

[14] Aboulghar MA Mansour RT Serour G II Ramzy AM Amin YM Mansour RT Oocyte quality in patients with severe ovarian hyperstimulation syndrome. Fertil Steril (1997) 68(6):1017–21. 10.1016/s0015-0282(97)00409-3 9418690

[15] Fábregues F Peñarrubia J Vidal E Casals G Vanrell JA Balasch J.Oocyte quality in patients with severe ovarian hyperstimulation syndrome: a self-controlled clinical study. Fertil Steril (2004) 82(4):827–33. 10.1016/j.fertnstert.2004.02.131 15482755

[16] Sahu B Ozturk O Ranierri M Serhal P Comparison of oocyte quality and intracytoplasmic sperm injection outcome in women with isolated polycystic ovaries or polycystic ovarian syndrome. Arch Gynecol Obstetr (2008) 277(3):239–44. 10.1007/s00404-007-0462-x 17899140

[17] Practice Committee of the American Society for Reproductive Medicine Prevention and treatment of moderate and severe ovarian hyperstimulation syndrome: a guideline. Fertil Steril (2016) 106(7):1634–47. 10.1016/j.fertnstert.2016.08.048 27678032

[18] Goodman NF Cobin RH Futterweit W Glueck JS Legro RS Carmina E American Association of Clinical Endocrinologists (AACE); American College of Endocrinology (ACE); Androgen Excess and PCOS Society (AES). AMERICAN ASSOCIATION OF CLINICAL ENDOCRINOLOGISTS, AMERICAN COLLEGE OF ENDOCRINOLOGY, AND ANDROGEN EXCESS AND PCOS SOCIETY DISEASE STATE CLINICAL REVIEW: GUIDE TO THE BEST PRACTICES IN THE EVALUATION AND TREATMENT OF POLYCYSTIC OVARY SYNDROME–PART 1. Endocr Pract (2015) 21(11):1291–300. 10.4158/EP15748.DSC 26509855

[19] Zivi E Simon A Laufer N Ovarian hyperstimulation syndrome: definition, incidence, and classification. Semin Reprod Med (2010) 28(6):441–7. 10.1055/s-0030-1265669 21082501

[20] Blumenfeld Z The Ovarian Hyperstimulation Syndrome. Vitam Horm (2018) 107:423–51. 10.1016/bs.vh.2018.01.018 29544639

[21] American College of Obstetricians and Gynecologists Committee on Gynecologic Practice and Practice Committee Female age-related fertility decline. Fertil Steril (2014) 101:633–4. 10.1016/j.fertnstert.2013.12.032 24559617

[22] Esteves SC Carvalho JF Martinhago CD Melo AA Bento FC Humaidan P Estimation of age-dependent decrease in blastocyst euploidy by next generation sequencing: development of a novel prediction model. Panminerva Med (2019) 61(1):3–10. 10.23736/s0031-0808.18.03507-3 29962186

[23] Ata B Kaplan B Danzer H Glassner M Opsahl M Tan SL Array CGH analysis shows that aneuploidy is not related to the number of embryos generated. Reprod BioMed Online (2012) 24:614–20. 10.1016/j.rbmo.2012.02.009 22503277

[24] Ashrafi M Bahmanabadi A Akhond MR Arabipoor A Predictive factors of early moderate/severe ovarian hyperstimulation syndrome in non-polycystic ovarian syndrome patients: a statistical model. Arch Gynecol Obstet (2015) 292:1145–52. 10.1007/s00404-015-3723-0 25920524

[25] Johnson MD Williams SL Seager CK Liu JH Barker NM Hurd WW Relationship between human chorionic gonadotropin serum levels and the risk of ovarian hyperstimulation syndrome. Gynecol Endocrinol (2014) 30:294–7. 10.3109/09513590.2013.875998 24455971

[26] Sousa M Cunha M Teixeira da Silva J Oliveira C Silva J Viana P Ovarian hyperstimulation syndrome: a clinical report on 4894 consecutive ART treatment cycles. Reprod Biol Endocrinol (2015) 13:66. 10.1186/s12958-015-0067-3 26100393 PMC4477314

[27] Ma T Niu Y Wei B Xu L Zou L Che X Moderate-to-severe ovarian hyperstimulation syndrome: A retrospective multivariate logistic regression analysis in Chinese patients. Adv Clin Exp Med (2020) 29(1):85–90. 10.17219/acem/92916 31990458

[28] Mathur RS Akande AV Keay SD Hunt LP Jenkins JM Distinction between early and late ovarian hyperstimulation syndrome. Fertil Steril (2000) 73:901–7. 10.1016/s0015-0282(00)00492-1 10785214

[29] Aramwit P Pruksananonda K Kasettratat N Jammeechai K Risk factors for ovarian hyperstimulation syndrome in Thai patients using gonadotropins for in vitro fertilization. Am J Health Syst Pharm (2008) 65:1148–53. 10.2146/ajhp070566 18541685

[30] Lainas GT Lainas TG Sfontouris IA Venetis CA Bosdou JK Chatzimeletiou A Association between body mass index and oocyte maturation in patients triggered with GnRH agonist who are at high risk for severe ovarian hyperstimulation syndrome: an observational cohort study. Reprod Biomed (2019) 40(1):168–75. 10.1016/j.rbmo.2019.10.006 31839394

[31] Cook CL Siow Y Taylor S Fallat ME Serum mullerian-inhibiting substance levels during normal menstrual cycles. Fertil Steril (2000) 73:859–61. 10.1016/s0015-0282(99)00639-1 10731554

[32] Ocal P Sahmay S Cetin M Irez T Guralp O Cepni I Serum anti-Müllerian hormone and antral follicle count as predictive markers of OHSS in ART cycles. J Assisted Reprod Genet (2011) 28(12):1197–203. 10.1007/s10815-011-9627-4 PMC3241835 21882017

[33] Aboulghar M Prediction of ovarian hyperstimulation syndrome (OHSS). Estradiol level has an important role in the prediction of OHSS. Hum Reprod (2003) 18(6):1140–1. 10.1093/humrep/deg208 12773437

[34] Mocanu E Redmond ML Hennelly B Collins C Harrison R Odds of ovarian hyperstimulation syndrome (OHSS) - time for reassessment. Hum Fertil (Camb) (2007) 10(3):175–81. 10.1080/14647270701194143 17786650

