# Supplementary material for: Factors Associated with Ovarian Hyperstimulation Syndrome (OHSS) Severity in Women With Polycystic Ovary Syndrome Undergoing IVF/ICSI
Source: Front Endocrinol (Lausanne). 2021 Jan 19;11:615957. doi: 10.3389/fendo.2020.615957 (PMC7851086; doi:10.3389/fendo.2020.615957)
Supplement: Supplementary Figure 1 — Flow chart of the patient selection process. [file Image_1.pdf]

Supplementary figures

Manuscript title: Factors associated with ovarian hyperstimulation syndrome (OHSS) severity in women with polycystic ovary syndrome undergoing IVF/ICSI

Authors: Sun Bo#, Ma Yujia#,Li Lu, Hu linli, Wang Fang, Zhang Yile, Dai Shanjun, Sun yingpu*

Supplement Figure 1. Flow chart of the women selection process.

Supplement Figure 2 Relationships between the OHSS and risk factors of (A) antral follicular count and (B) basal FSH.

Supplement Figure 1. Flow chart of the women selection process.

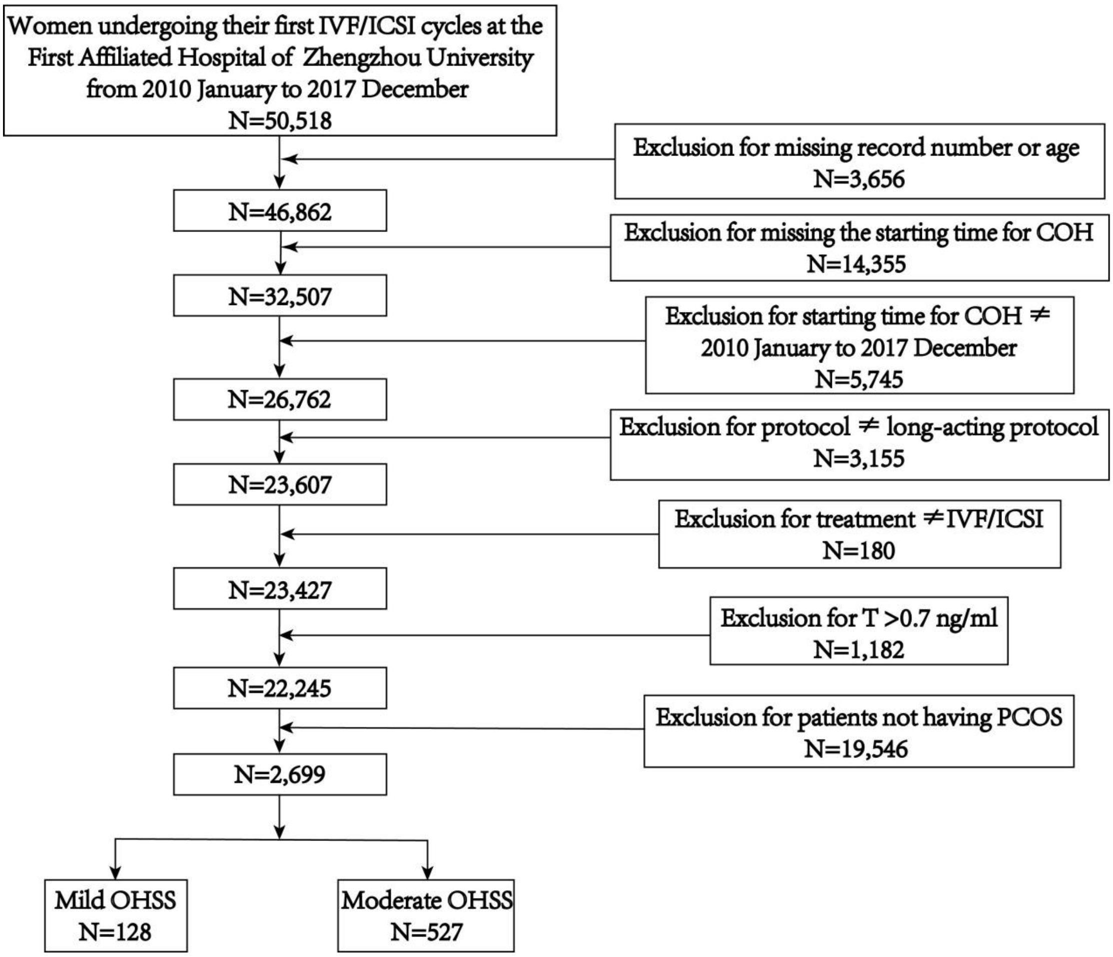

Supplement Figure 2 Relationships between the OHSS and risk factors of (A) antral follicular count and (B) basal FSH.

A. B.

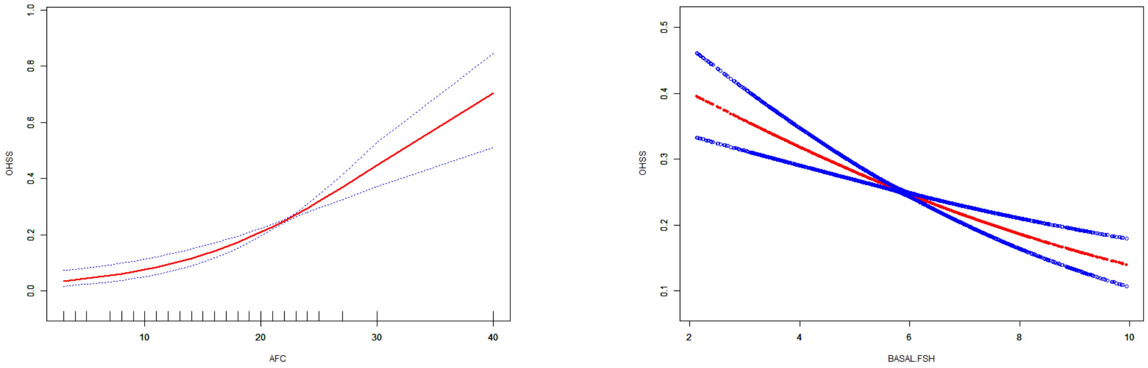

Note:

A. Adjusted for age, BMI, and basal FSH. B. Adjusted for AFC, Age, and BMI.
